# PC-1 works in conjunction with E3 ligase CHIP to regulate androgen receptor stability and activity

**DOI:** 10.18632/oncotarget.13230

**Published:** 2016-11-09

**Authors:** Jian Wang, Hui Zhang, Xiaoqing Zhang, Peng Wang, Hongtao Wang, Fang Huang, Chenyan Zhou, Jianguang Zhou, Shanhu Li

**Affiliations:** ^1^ Laboratory of Medical Molecular Biology, Beijing Institute of Biotechnology, Beijing 100850, China; ^2^ School of Medicine, Tsinghua University, Beijing 100084, China

**Keywords:** androgen receptor, prostate cancer, PC-1, CHIP, protein degradation

## Abstract

The androgen receptor (AR) is not only a ligand-dependent transcription factor, but also functions as a licensing factor, a component of DNA replication, which is degraded during mitosis. Furthermore, the deregulation of AR activity is involved in the initiation of prostate cancer and contributes to castration resistant prostate cancer (CRPC). While AR degradation is known to occur primarily through a proteasome-mediated pathway, very little is known about how this process is regulated, especially in M phase. PC-1 is an androgen-responsive factor and expresses specificity in prostate cancer, with higher expression noted at G2/M. In this study, PC-1 was shown to interact with AR and E3 ligase CHIP (Carboxy-terminus of Hsc70 Interacting Protein) and to enhance AR/CHIP interactions, thereby decreasing AR stability. Moreover, PC-1 was found to act in conjunction with CHIP in the decreasing of AR via ubiquitination, with the subsequent degradation predominantly occurring during M phase. PC-1 was also found to repress AR transcriptional activity in androgen-dependent and androgen-independent prostate cancer cells and attenuate the growth inhibition of AR. In conclusion, these findings should provide new clues regarding the modulation of AR turnover and activity via PC-1 and reveals an essential role of PC-1 in AR signaling.

## INTRODUCTION

The androgen receptor (AR) is a ligand-dependent transcription factor belonging to the steroid hormone receptor superfamily and consists of three functional domains: the N-terminal transactivation domain, the C-terminal DNA-binding domain (DBD) and the ligand-binding domain (LBD) [1, 2]. Upon binding to androgen, the AR translocates into the nucleus and recruits basic transcription machinery, to include cofactors regulating androgen-responsive gene transcription [3]. In addition to functioning as a transcription factor, experimental observations suggest that AR is also a licensing factor for AR-positive and androgen-sensitive prostate cancer cells and associates with licensing proteins Orc2, Cdc-6, Cdt-1 and Mcm2 [4–6] .

Deregulation of the AR signaling pathway, most likely through AR, is associated with the formation and development of primary prostate cancer as well as castration resistant prostate cancer (CRPC) progression [7–9]. Abnormal AR functions, such as functional activation, are increasingly recognized in prostate cancer development and progression, with large bodies of evidence indicating that AR activities and levels are precisely modulated by many cofactors at both transcriptional and post-translational levels. AR activity can be modulated by post-translational modifications (PTMs), such as phosphorylation, acetylation, SUMOylation and ubiquitination [10–14]. It has been reported that AR is stabilized by the S26 proteasome inhibitor MG-132, suggesting that AR is targeted for degradation through this proteasome pathway. As a licensing factor, AR must be degraded during mitosis in order to allow DNA replication to reinitiate for subsequent cell cycling, with AR stabilization during mitosis inhibiting prostate cancer proliferation [5]. A number of E3 ligases, Mdm2 [13], CHIP [15, 16], Nedd4 [17], SPOP [18] and Siah2 [19], have been discovered to control AR stability and activity. While AR degradation is known to occur primarily through a proteasome-dependent pathway, very little is known about how this process is regulated, especially during M phase.

PC-1, also named PrLZ, belongs to the TPD52 gene family. PC-1 and TPD52 share homologous C-terminal domains, including a coiled-coil leucine zipper. Distinctively, PC-1 possesses a specific N-terminal 1-46 amino acid domain and its own transcription regulatory element. Moreover, PC-1 expression is predominantly prostate tissue specific and androgen responsive, whereas TPD52 expression can be detected in many tissue and cell types and is not affected by androgen [20, 21]. PC-1 expression is significantly associated with clinical prostate cancer progression and is positively correlation with prostate cancer cell growth. Furthermore, PC-1 expression was found to promote androgen-independent progression and Casodex resistance, an androgen antagonist, in prostate cancer cells [22]. In this report, we show that PC-1 can interact with AR and E3 ligase CHIP (Carboxy-terminus of Hsc70 Interacting Protein), while at the same time enhance AR interactions with CHIP and regulating AR stability, especially AR degradation during M phase of the cell cycle.

## RESULTS

### PC-1 regulates AR protein stability

**Figure 1:**
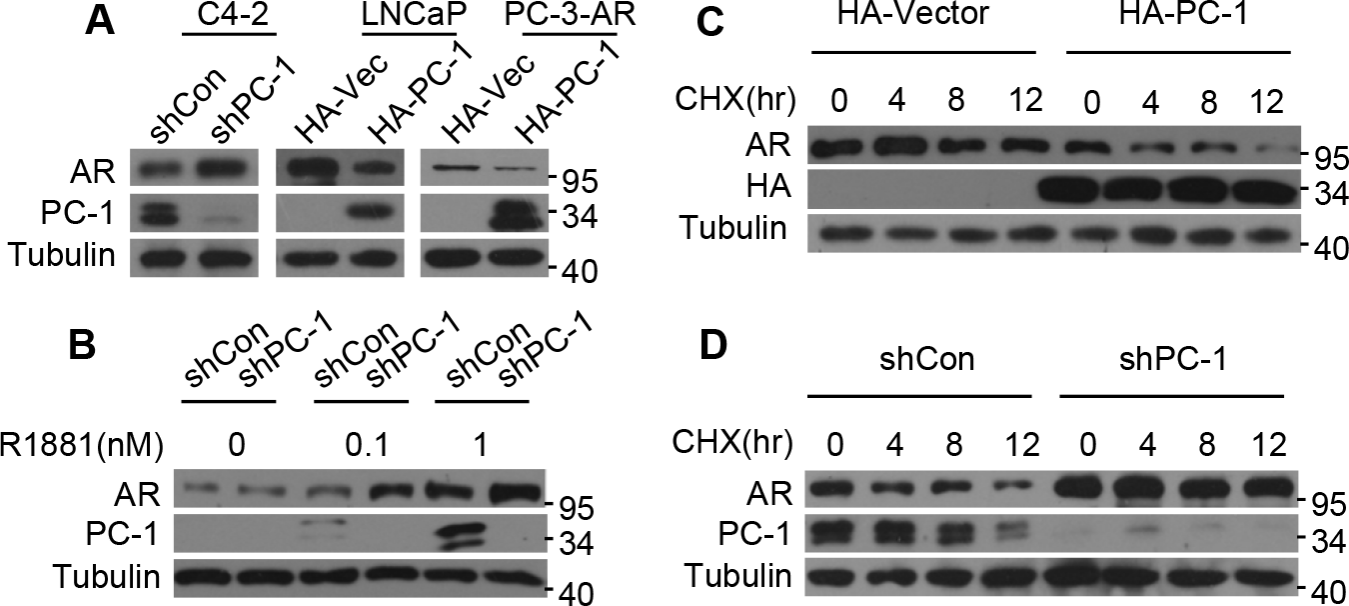
PC-1 regulates AR protein stability (**A**) C4-2 cells infected with lentiviruses encoding shRNAs for control vector (shCon) or PC-1 (shPC-1), LNCaP cells and PC-3-AR cells stably transfected with control PCDNA-HA or PCDNA-HA-PC-1. Cells were cultured in whole medium and cell lysates were analyzed for AR and PC-1 levels by Western blotting. (**B**) PC-1 knockdown C4-2-shPC-1 and control C4-2-shCon cells were cultured in androgen-deprived medium with different concentration of androgen R1881. AR and PC-1 protein levels were analyzed by Western blotting. (**C**) PC-1 over-expressing LNCaP cells and (**D**) PC-1 knockdown C4-2 cells were treated with 10 uM CHX and then lysates were collected at the indicated times for Western blotting.

To investigate the relationship between PC-1 and AR in prostate cancer progression, we explored the possibility that PC-1 regulates AR protein stability. Because PC-1 was identified to be up-regulated in C4-2 in comparison with LNCaP parent cell line and did not express in PC-3 cell line, we overexpressed PC-1 in LNCaP cells and PC-3-AR cells and knocked down PC-1 in C4-2 cells. As shown in Figure 1A, knockdown PC-1 increased AR protein levels in C4-2 cells while PC-1 overexpression could reduce AR protein levels in LNCaP and PC-3-AR cells. To further verify these observations, PC-1 expression was knocked down in C4-2 cell line, with an inverse correlation between PC-1 and AR noted when cells were cultured in medium with different concentration of androgen R1881 (Figure 1B). To ascertain whether PC-1 could accelerate AR protein levels turnover, cells were treated with cycloheximide (CHX) to block protein synthesis and measured the half-life of the AR protein. In LNCaP cells, PC-1 overexpression dramatically decreased the AR protein half-life, while PC-1 knockdown in C4-2 cells increased the half-life of endogenous AR (Figure 1C, 1D). These results indicate that PC-1 may regulate AR protein level by promoting AR degradation.

### PC-1 enhances AR interactions with E3 ligase CHIP

**Figure 2:**
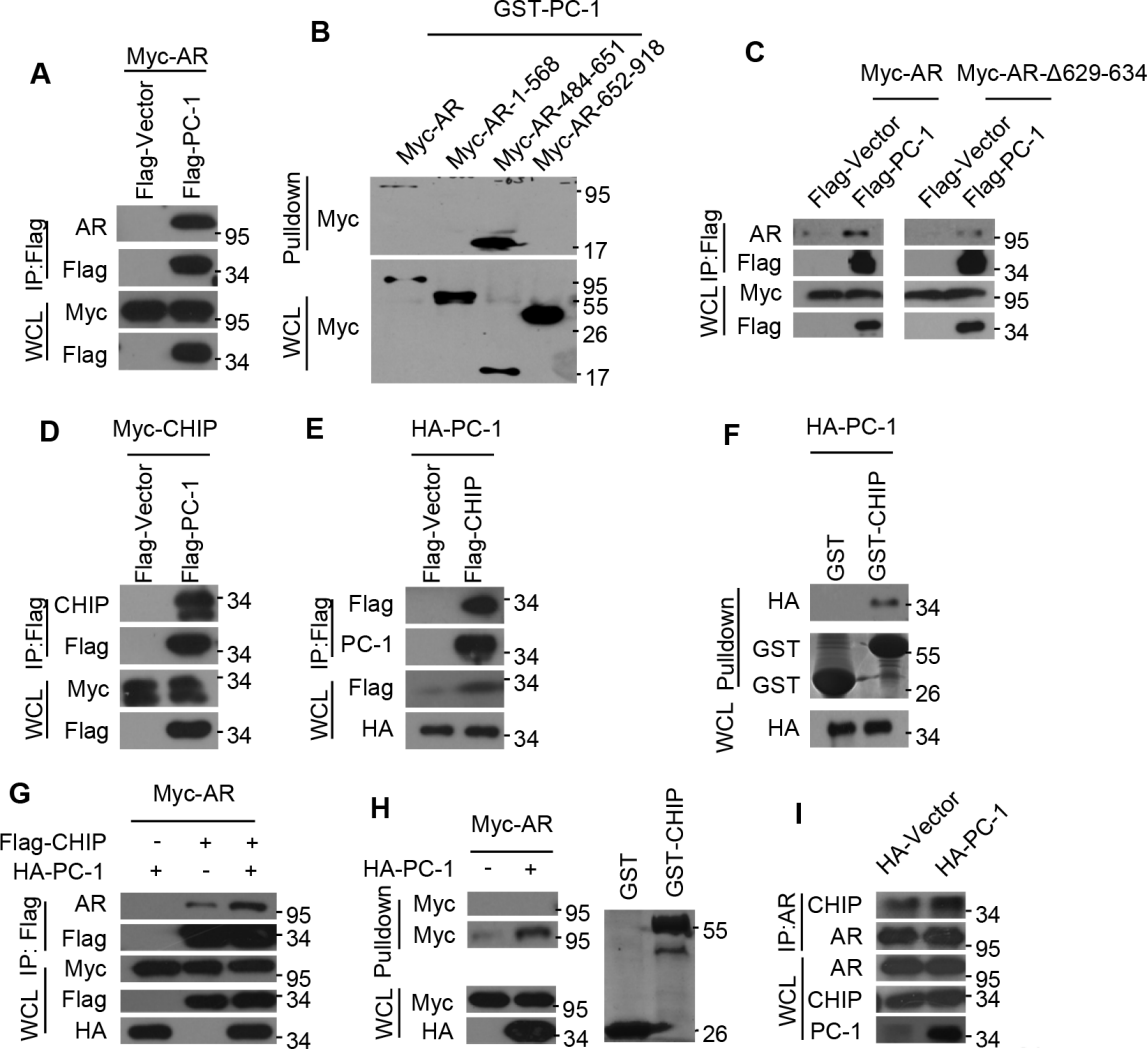
PC-1 enhances AR and E3 ligase CHIP interactions (**A**) Myc-AR was expressed in HEK293 cells either alone or together with Flag-PC-1. Lysates were immunoprecipitated with anti-FLAG antibody and immunoblotted with the indicated antibodies for Western blotting. (**B**) Myc-AR and AR deletion mutants were constructed and expressed in HEK293 cells independently. Lysates were incubated with purified GST-PC-1 for 4–6 h, separated by PAGE and immunoblotted with anti-Myc antibody for Western blotting. (**C**) HEK293 cells were cotransfected with Flag-PC-1 or vector either with Myc-AR or its mutants ARΔ629-634. Lysates were immunoprecipitated with anti-FLAG antibody and immunoblotted with the indicated antibodies for Western blotting. (**D**) Myc-CHIP and Flag-PC-1 or (**E**) HA-PC-1 and Flag-CHIP were expressed in HEK293 cells either independently or jointly. Lysates were immunoprecipitated with anti-FLAG antibody and immunoblotted with the indicated antibodies for Western blotting. (**F**) HA-PC-1 were expressed in HEK293 cells and lysates were pulled-down with GST-CHIP or GST protein and immunoblotted with anti-HA antibody for Western blotting. (**G**) Myc-AR was expressed in HEK293 cells either with HA-PC-1 or Flag-CHIP alone or with both together. Lysates were immunoprecipitated with anti-FLAG antibody and immunoblotted with the indicated antibodies for Western blotting. (**H**) Myc-AR and HA-PC-1 were expressed in HEK293 cells either independently or jointly. Lysates were incubated with purified GST or GST-CHIP for 4-6 h, separated by PAGE and immunoblotted with anti-Myc antibody for Western blotting. Left: upper panel is the purified GST incubated with lysates and lower is the purified GST-CHIP incubated with lysates; Right: Gel was Coomassie Blue-stained to show relative loading of recombinant GST and GST-CHIP. (**I**) Lysates of LNCaP cells and PC-1 overexpressing LNCaP-PC-1 cells were immunoprecipitated with anti-AR antibody and immunoblotted with the indicated antibodies for Western blotting.

Having seen that PC-1 can regulate AR protein stability, we subsequently examined whether these two proteins could interact via co-immunoprecipitations *in vivo*. HEK293 cells were transfected with both PC-1 and AR expressing plasmids, with exogenous AR readily detected in exogenous PC-1 pull-down complexes (Figure 2A). To map the specific AR region required for PC-1 interaction, several AR deletion mutants were constructed and expressed in HEK293 cells. GST-pulldown assays were performed to confirm that PC-1 could interact with wt-AR and AR-484-651 (Figure 2B). Due to the presence of a hinge region between AR DBD and LBD and this region's interactions with several proteins [23], we wanted to determine if PC-1 could interact with this region. A subsequent Co-IP experiment confirmed that the ARΔ629-634 mutant reduced PC-1 interactions when compared with wt-AR (Figure 2C). This observation suggests that PC-1 predominantly interacts with the AR hinge region.

The CHIP is an ubiquitin E3 ligase that has been shown to interact with AR and target it for proteasomal degradation [15, 16]. Since PC-1 interacts with AR and also promotes its degradation, we wanted to determine whether the actions of PC-1 are correlated with CHIP. To verify this possibility, Myc-CHIP was expressed with or without Flag-PC-1 in HEK293 cells, with lysates subjected to immunoprecipitation with antibodies against Flag-PC-1. Western blot analysis of the immunoprecipitates revealed specific Co-IP of CHIP with PC-1 and vice versa (Figure 2D, 2E). To further support this finding, a GST pull-down experiment was performed using lysates from HEK293 cells overexpressing HA-PC-1 and incubated with GST-CHIP or GST protein. The GST-CHIP was able to pull-down PC-1, thus confirming that PC-1 could interact with CHIP E3 ligase (Figure 2F). Since PC-1 was found to interact with both AR and CHIP, the ability of PC-1 to modulate AR and CHIP interactions was examined. As shown in Figure 2G, Co-IP revealed that PC-1 overexpression enhances CHIP and AR interactions, thus further substantiating the GST-pulldown assay results, with GST-CHIP able to pull-down more AR protein when incubated with HEK293 cell lysates cotransfected with PC-1 (Figure 2H). Furthermore, PC-1 was shown to enhance endogenous AR and CHIP interactions. When AR was immunoprecipitated from LNCaP cells or overexpressing PC-1 LNCaP stable cells, higher levels of endogenous CHIP/AR complexes were noted in cells overexpressing PC-1 (Figure 2I).

### PC-1 promotes AR ubiquitination and degradation in a CHIP-mediated manner

**Figure 3:**
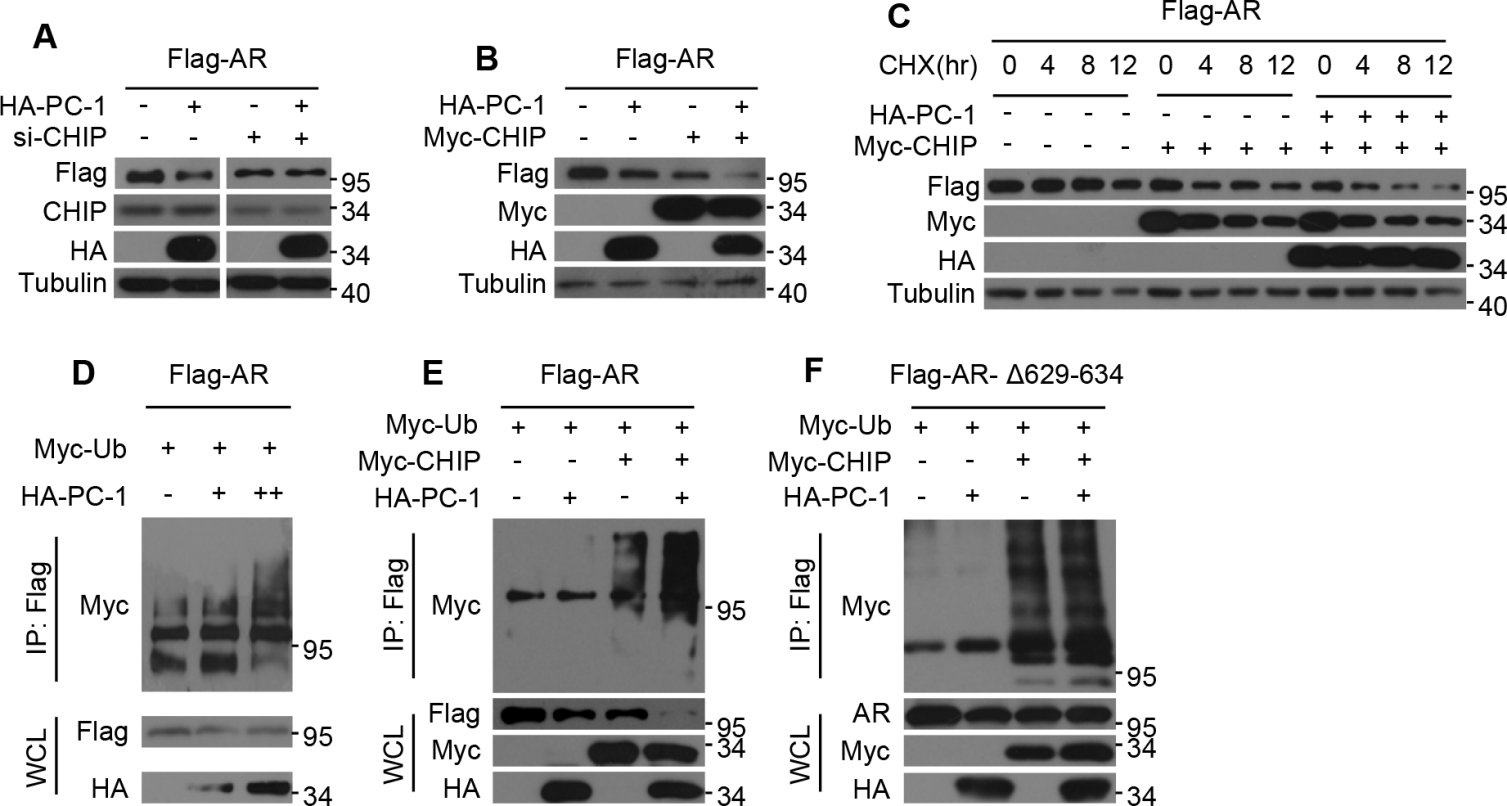
PC-1 promotes CHIP-mediated AR ubiquitination and degradation (**A**) Flag-AR and HA-PC-1 were expressed either alone or together in HEK293 cells which were transfected with scrambled siRNA or CHIP siRNA. Lysates were prepared and immunoblotted with the indicated antibodies for Western blotting. (**B**) Flag-AR was expressed in HEK293 cells either with HA-PC-1 or Myc-CHIP alone or with both together. Lysates were immunoblotted with the indicated antibodies for Western blotting. (**C**) Flag-AR was expressed in HEK293 cells alone or with Myc-CHIP or with Myc-CHIP and HA-PC-1 together. HEK293 cells were treated with 10 uM CHX and lysates were collected at the indicated times for Western blotting. (**D**) Flag-AR and Myc-ubiquitin plasmads were co-expressed with different amounts of HA-PC-1. (**E**) Wild type Flag-AR or (**F**) hinge region mutant Flag-ARΔ629-634 and Myc-ubiquition plasmads were expressed in HEK293 cells either with HA-PC-1 or Myc-CHIP independently or with both jointly. 36 h after transfection, Lysates were immunoprecipitated with anti-FLAG antibody and immunoblotted with anti-Myc antibody for Western blotting.

Our previous work found that PC-1 could interact with AR and E3 ligase CHIP and enhance their interactions. We also showed that PC-1 could down-regulate AR protein stability, thus suggesting that perhaps PC-1 regulation of AR stability is dependent on E3 CHIP. To confirm this hypothesis, we knocked down endogenous CHIP in HEK293 cells and found that AR protein levels remained unchanged even after cotransfection with the PC-1 (Figure 3A). Next, the effects of PC-1 on CHIP-mediated degradation and the AR turnover rate were examined. In HEK293 cells, the co-overexpression of PC-1 with CHIP resulted in a significant decrease in protein levels and the AR half-life when compared with the overexpression of PC-1 or CHIP alone (Figure 3B, 3C). Therefore, PC-1 and CHIP jointly attenuated AR stability. Due to the fact that AR proteasome degradation requires ubiquitination, the potential role of PC-1 in AR ubiquitination was examined. In HEK293 cells, Myc-ubiquitin-dependent AR-ubiquitination was enhanced by the overexpression of PC-1 protein (Figure 3D). Next, the ability of PC-1 to regulate AR ubiquitination through CHIP E3 ligase was examined. Co-expression of PC-1 with CHIP increased AR ubiquitination in HEK293 cells compared with transfection with CHIP alone (Figure 3E), while the ubiquitination of the mutant AR-Δ629-634, which cannot interact with PC-1, did not change. All of these findings indicate that PC-1 promotes AR ubiquitination and degradation in an E3 ligase CHIP-mediated manner.

### PC-1 and CHIP regulate AR degradation in M phase

**Figure 4:**
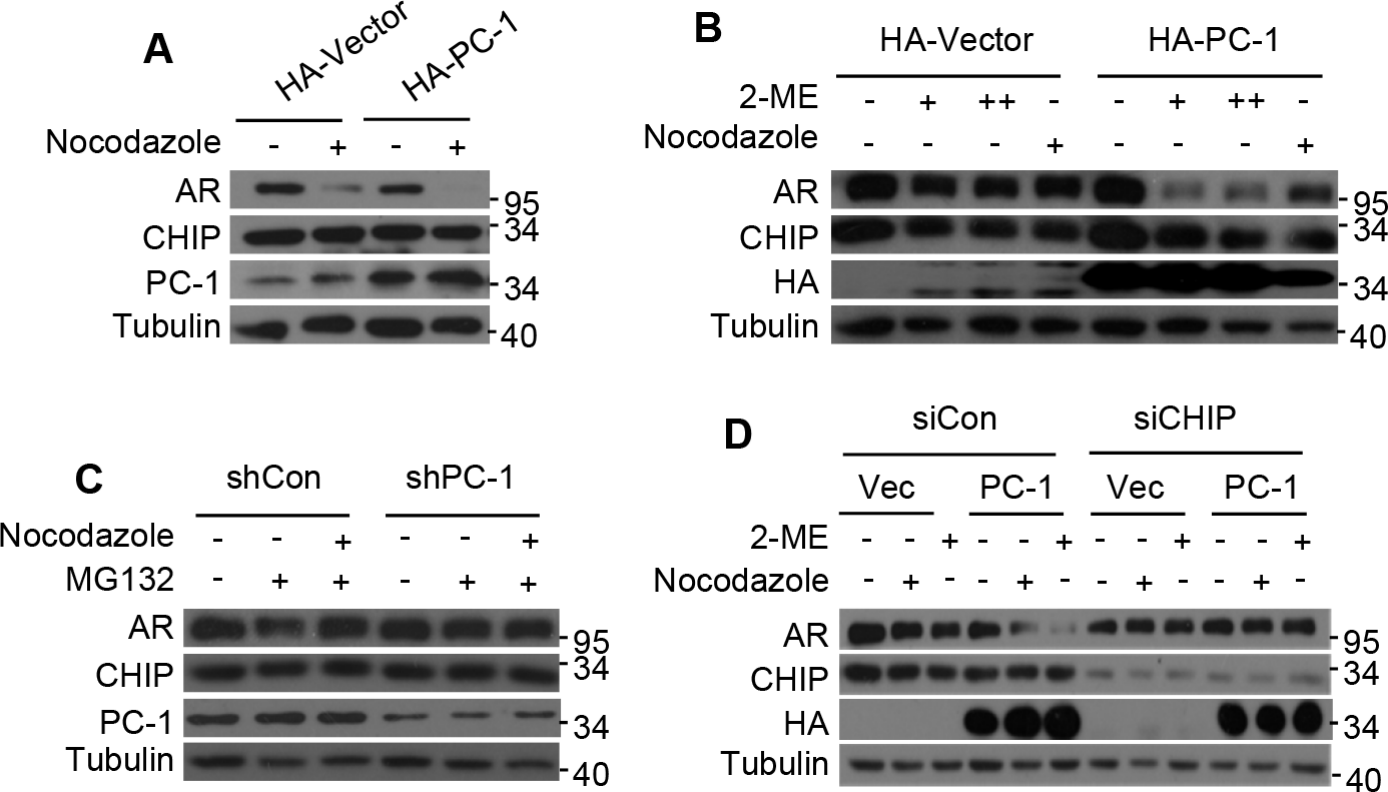
PC-1 works in conjunction with CHIP to regulate AR degradation in M phase (**A**) LNCaP cells and PC-1 overexpressing LNCaP stable cells were arrested in M phase 16 h post-treatment with 200 ng/ml nocodazole or (**B**) 24 h post-treatment with 2 uM or 5 uM 2-ME. (**C**) C4-2 cells and PC-1 knockdown C4-2 stable cells were treated with 200 ng/ml nocodazole for 16 h, with or without proteasome inhibitor MG132 (5 mg/ml). Proteasome inhibitor MG132 was added 6 h before cell lysates were collected. (**D**) LNCaP cells and PC-1 overexpression LNCaP stable cells with endogenous CHIP knockdown or without knowdown were treated with 200 ng/ml nocodazole for 16 h or treated with 2 uM 2-ME for 24 h. (A)–(D) Lysates were immunoblotted with the indicated antibodies for Western blotting.

Previous studies have shown that AR acts as a DNA licensing factor and should be degraded in M phase of the cell cycle to allow initiation of the next cell cycle, while PC-1 and D53 expression, both members of the tumor protein D52 family, have been reported to increase at the G2-M transitions [24, 25]. To determine whether decreased AR expression coincides with PC-1 expression in M phase, nocodazole or 2-Methoxyestradiol (2-ME) were used to arrest LNCaP cells at G2/M phase. AR expression level decreased significantly in M phase and PC-1 overexpression further induced AR degradation (Figure 4A, 4B). In C4-2 cells, endogenous PC-1 expression was knocked down and cells were arrested in M phase using nocodazole, with nocodazole-induced AR degradation reduced (Figure 4C). Furthermore, E3 ligase CHIP knockdown in LNCaP cells that were arrested in M phase with nocodazole or 2-ME showed that AR down-regulation was effectively inhibited even with PC-1 overexpression (Figure 4D). These findings demonstrated that PC-1 is involved in AR degradation in M phase, with this degradation dependent on E3 CHIP activity.

### PC-1 inhibits AR transcriptional activity and attenuates AR suppression on prostate cancer cell growth

**Figure 5:**
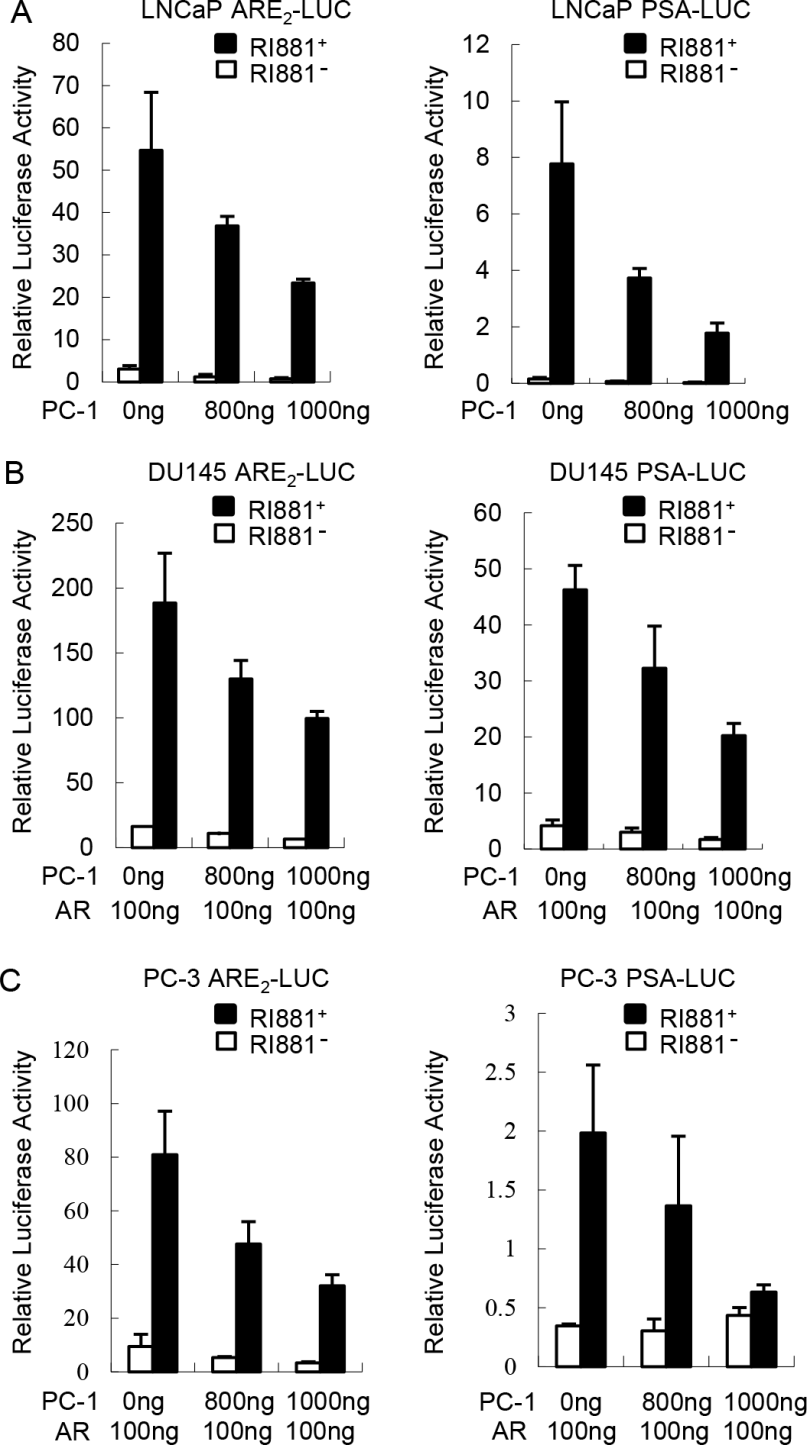
PC-1 inhibits AR transcriptional activity (**A**) LNCaP cells were transiently cotransfected with 300 ng ARR2-Luciferase or PSA6.1-Luciferase reporter plasmids with 800 ng or 1000 ng pcDNA3.1-hPC-1 plasmids and 25 ng of pRL-TK Renilla reporter. After transfection 36 h, the medium was replaced with steroid-free medium in the presence or absence of 1 nM R1881 for 12 h. The lysates were harvested 48 h after transfection and the luciferase activity was measured using commercially available kits from Promega. (**B**) DU145 and (**C**) PC-3 cells were transiently transfected with AR and other expression plasmids as indicated. The luciferase activity was measured and normalized as described in panel A. Data shown represent three independent experiments.

Since PC-1 in conjunction with E3 ligase CHIP accelerate AR degradation, we wanted to determine whether PC-1 affects AR transcriptional activity. The effect of PC-1 overexpression on a probasin promoter based ARR2-Luciferase or PSA promoter based PSA6.1-Luciferase reporter was examined in LNCaP cells. The luciferase assay revealed that PC-1 was capable of suppressing AR transcriptional activity whether stimulated with the R1881 or not (Figure 5A). Additionally, PC-1 showed the same suppression of AR transcriptional activity in AR negative PC-3 and DU-145 cell lines when transiently transfected with AR expressing plasmids (Figure 5B, 5C). These results indicated that PC-1 can both suppress endogenous and ectopically expressing AR transcriptional activity.

**Figure 6:**
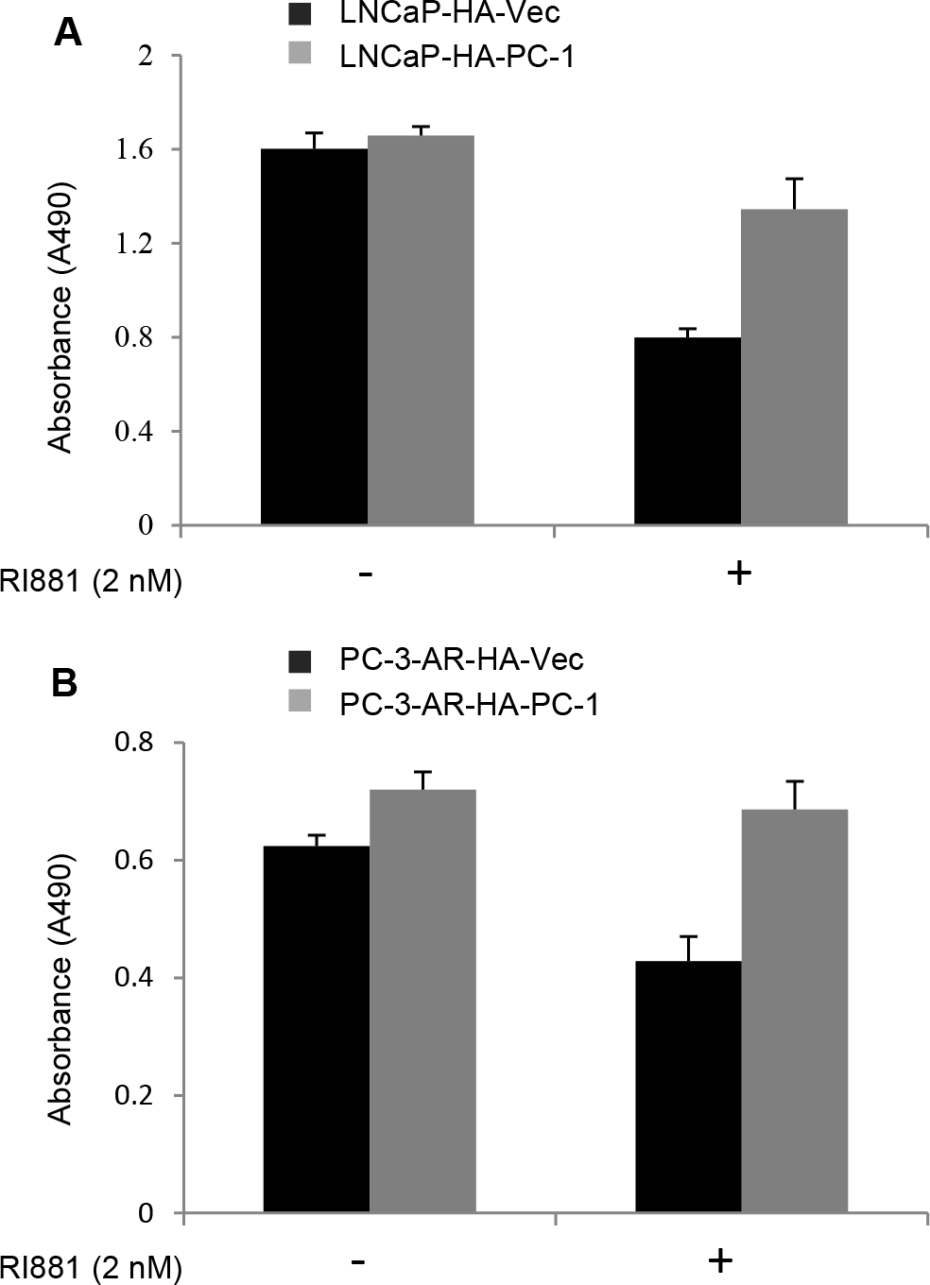
PC-1 attenuates AR suppression on prostate cancer cell growth (**A**) LNCaP-HA-PC-1 and control LNCa-HA-Vector cells and (**B**) PC-3-AR-HA-PC-1 and control PC-3-AR-HA-Vector cells were seeded in 96-well plate at confluence of 2000 cells per well in 200 μl RPMI 1640 containing 10% FBS with or without 2 nM R1881 treatment. 7 days later, the growth of cells (indicated by absorbance at 490 nM) was measured with the MTT method. Assays were repeated 3 times, and error bars represent SD.

AR over-activation at high concentration of androgen has been reported to inhibit prostate cancer cell growth [35]. In order to determine whether PC-1 suppressing AR protein stability and transcriptional activity can attenuate AR inhibition on prostate cancer cell growth stimulated with R1881, MTT assay was performed. PC-1 overexpression LNCaP /PC-3-AR cells and control LNCaP/PC3-AR cells were cultured in media containing 10% FBS with and without 2 nM R1881 for 7 days. As shown in Figure 6A, 6B, the control LNCaP and PC-3-AR cells showed significant growth inhibition after stimulation with 2 nM R1881, while PC-1 overexpression LNCaP and PC-3-AR cells were less inhibited with 2 nM R1881 stimulation and proliferation is faster than control cells. These results showed that AR can suppress prostate cancer cells proliferation at high concentration of androgen while PC-1 expression can attenuate the growth inhibition of AR.

## DISCUSSION

Deregulation of the AR signaling pathway is crucial for prostate cancer cell proliferation, tumor progression and the development of CRPC. Understanding the mechanisms underlying the regulation of AR activity is important for the development of effective therapeutic modalities to treat CRPC. Various posttranslational modifications including phosphorylation, acetylation, SUMOylation and ubiquitination have been shown to regulate AR expression, stability and activity under various cellular conditions, with accumulating evidence indicates that AR can be targeted for degradation vis ubiquitin-proteasome pathways. In this report, we have demonstrated that PC-1 forms a complex with AR and E3 ubiquitin ligase CHIP, at the same time enhancing AR and CHIP interactions and regulating CHIP-mediated AR stability, especially AR degradation in M phase of the cell cycle.

In this report, PC-1 overexpression was found to decrease AR expression, whereas endogenous PC-1 knockdown increased AR expression, thus indicating that PC-1 is involved in the regulation of AR stability. Previous studies have also identified some cofactors that play a role in modulating AR stability via the ubiquitin-proteasome pathway. For example, MDM2 E3 ubiquitin ligase targets AR degradation via AKT mediated phosphorylation [13]. PMEPA1, as an androgen-responsive gene, functions as a negative regulator of AR by recruiting the Nedd4 ubiquitin E3 ligase to AR [17]. LncRNA HOTAIR binds the NTD of the AR protein and consequently blocks the recruitment of MDM2, thus protects AR from ubiquitin-mediated degradation [26]. In this report, Co-IP and GST-pulldown both confirmed that PC-1 can interact with AR and E3 ligase CHIP, with PC-1 enhance AR/CHIP interactions. This suggests that PC-1 may serve as a scaffold to recruit CHIP and AR. Furthermore, PC-1 overexpression was shown to increases CHIP-mediated AR ubiquitination, with CHIP knockdown abrogating PC-1 mediated AR degradation. Therefore, PC-1 may act in conjunction with CHIP to decrease AR stability. This research also maps the hinge region of AR, which interacts with PC-1, and showed that a deletion at AR 629-634 of the hinge region abolished PC-1 and AR interactions and the PC-1 mediated enhancement of CHIP-mediated AR ubiquitination. Recent results indicate that the hinge region of AR is a phosphorylation and acetylation target site and an interaction domain for several proteins [27]. Furthermore, it is a multifunctional region involved in DNA binding, nuclear localization and transactivational modulation [23]. Increasing evidence suggests that the expression of AR variants is upregulated during castration-resistant progression of prostate cancer and that increased AR variant expression may contribute to anti-androgen therapy resistance [28, 29, 30]. The majority of the AR variants identified thus far do not harbor the hinge domain and most importantly it has been reported that AR variants that lack the hinge region can activate their transcription activity independent of androgen, thus possibly potentiating the CRPC phenotype [18, 23, 31]. This implies that the CRPC associated increased PC-1 expression may not respond due to AR variants that lack the hinge region being able to potentially escape PC-1-mediated degradation, thus maintaining high transcriptional activity and contributing to prostate cancer progression.

Another important finding of our study is that PC-1 increases AR degradation in M phase in coordination with E3 ligase CHIP. Besides being a transcription factor, AR has a potential role in DNA licensing for AR-expressing prostate cancer cells. As a licensing factor, AR needs to be removed from the DNA and degraded in mitosis or early-G phase for effective DNA relicensing since the lack of such degradation will result in the inhibition of cellular proliferation. While it was discovered that AR protein is degraded via the ubiquitin-proteasome in mitosis, very little is known about the mechanism of its degradation. Previous studies have shown that 2-ME, a naturally occurring estrogen metabolite, induces mitotic arrest in prostate cancer cells, thus activating CHIP and degrading AR [32]. Our research also confirms that when using nocodazole or 2-ME to arrest prostate cancer cell at G2/M phase, PC-1 overexpression reduces AR expression levels, while duel PC-1/CHIP knockdown protects AR from nocodazole or 2-ME induced degradation. This result suggests that PC-1 may play a role in modulating AR degradation during mitosis and also coincides with our previous research that showed that PC-1 expression increases during M phase in prostate cancer cells. Our result may not exclude other mechanisms that modulate AR degradation during M phase. 2-ME treatment can also active E3 ligase MDM2 [32] and PIM-1 kinase isoform PIM-1S, which increase in G2 and M-phase can induce Ser-213 phosphorylation of AR, enhancing AR/MDM2 interactions and modulating AR degradation during mitosis [33].

PC-1 can decrease AR stability through a CHIP-mediated proteasome pathway, with PC-1 shown to inhibit AR transcriptional activity using a luciferase assay. PC-1 is an androgen responsive gene and represses AR transcriptional activity via an apparent negative feedback mechanism. AR has emerged as a key molecular determinant in the progression of human prostate cancer and is a preferential treatment for men with metastatic prostate cancer, thus the finding that PC-1 is also associated with prostate cancer progression seems paradoxical. We think one explanation is that because PC-1 interacts with the hinge region of AR, the AR splice variants in which the hinge region is deleted or truncated can escape PC-1 regulation. Indeed, the deletion of residues ^629^RKLKKL^634^ of the hinge region unexpectedly resulted in AR proteins with much higher activity potency [23]. To date, 11 different AR-hinge region mutants in prostate cancer tissues or prostate cancer cell lines have been identified [27, 34]. Another explanation is that even though PC-1 down-regulates AR activity, it still promotes the progression of prostate cancer. More recent clinical outcomes demonstrate that in some patients diagnosed with CRPC, AR expression is lost, thus implying that diminished AR expression is associated with disease progression. High concentration of androgen has been reported to suppress proliferation of prostate cancer cells [35, 36]. In our work, we found that 2 nM R1881 suppressed growth of LNCaP and PC-3-AR cells and PC-1 expression attenuated the growth inhibition of AR, maybe the reason is that PC-1 inhibit AR transcriptional activity. Furthermore, PC-1 inhibits AR activity and at the same time promotes prostate cancer progression through a negative feedback mechanism. Our previous findings showed that PC-1 can increase AKT activity to promote prostate cancer progression, but the mechanism was unclear. Carver and colleagues showed that AR and AKT signaling pathways cross-regulate each other by reciprocal feedback, with an AR blockade relieving feedback inhibition of AKT by the phosphatase PHLPP [37]. PC-1 may also act by blocking AR activity to increase AKT activity and promote prostate cancer progression.

We noticed Li *et al* reported that PC-1 enhance AR transcriptional activity in the castration-resistant condition [38]. This finding is significant difference in our results. We cannot fully explain the possible causes, but we noticed their previous reports that they did not use PC-1 specific siRNA which could also knockdown TPD52 in C4-2 cells, so the function they found perphaps is not PC-1 specific; Their report also showed that PC-1 enhanced AR transcriptional activity and increased PC3-AR9 cell proliferation. But AR is a tumor suppressor in the prostate cancer cell line PC3-AR9 *in vitro* and *in vivo* [36, 39, 40], if PC-1 enhance AR transcriptional activity, it should inhibit PC-3-AR9 growth. We postulated such events may contribute to the discrepancy of the data reported by Li *et al* with our results.

In this report, we found a new clue regarding AR stability modulation via PC-1 acting in conjunction with E3 ligase CHIP. Further investigations on the pathophysiologic effects of PC-1 on AR function may reveal that PC-1 can serve as a valuable target in controlling prostate cancer progression.

## MATERIALS AND METHODS

### Expression plasmids

PC-1 plasmids was constructed by cloning PC-1 into pcDNA3.1-HA/Myc or pCMV-Flag 2B vector (kindly gifted from Dr Wannian Yang, Weis center for research, Geisinger Clinic); A series of AR expression plasmids encoding of AR (full length), AR-TAD (1-568) and DBD (484-651), LBD (652-918) and ARΔ629-634 were constructed by cloning corresponding AR fragments into pcDNA3.1-Myc or pCMV-Flag 2B. CHIP plasmids were constructed by cloning CHIP into pcDNA3.1-Myc or pCMV-Flag 2B vector; pARE_2_-Luc containing two copies of natural ARE and pSG5-hAR were gifts from Dr. Qinong Ye (Beijing Institute of Biotechnology); The PSA reporter luciferase construct was a gift from Dr. Leland W.K. Chung (Cedars-Sinai Medical Center); The bacterial expression vectors encoding full-length GST-PC-1 or GST-CHIP fusion proteins were constructed by cloning the corresponding fragments into pGEX-4T1 vector.

### Cell culture and drug treatments

HEK293, PC3 and DU145 cells were cultured in DMEM (Gibco) media with 10% fetal bovine serum (FBS, Hyclone) and 1% penicillin/streptomycin; LNCaP and C4-2 cell lines were cultured in RPMI 1640 (Gibco) with 10% FBS. All cells were cultured at 37°C with 5% CO_2_ in a humidified incubator. LNCaP-Zero and LNCaP-PC-1 were generated from LNCaP cells as described previously [22]. Human prostate cancer PC3 cells was stably transfected with pcDNA3.1-Myc-hAR and selected neomycin-resistant cells by incubation with 500 ug G418/ml.

To study the effect of androgen, the cells were treated with a synthetic androgen R1881 (Perkin-Elmer Life and Analytical Sciences) in fresh phenol red-free RPMI 1640 with 5% charcoal stripped fetal bovine serum (cFBS, Hyclone) for 12 h.

To arrest prostate cancer cells in M phase, the cells were treated with 200 ng/ml nocodazole (Sigma-Aldrich) for 16 h or 2 μM or 5 μM 2-ME (Sigma-Aldrich) for 24 h before lysed.

To test the AR turnover rate, the cells were treated with 10 uM CHX and were lysed at different time points for Western blotting.

### Reagents

Proteasome chemical inhibitor MG132 was from Selleck; Protein synthesis inhibitor CHX was from Calbiochem; Anti-Flag M2 affinity gel was from Sigma-Aldrich; Glutathione Sepharose 4B was from Amersham Biosciences; Anti-AR (sc-7305), anti-CHIP (sc-133066), anti-Myc (sc-40), anti-HA (sc-805), anti-Flag (sc-807) and anti-β-tubulin (sc-5274) antibodies were from Santa Cruz; Anti-PC-1 antibody which is against PC-1 N-terminal 46 amino acids residues was made by our laboratory.

### shRNA and siRNA treatments

PC-1 knockdown construct was created by inserting annealing oligos (Forward oligo: CCGGAAGCTATCTCTACTTGTCTCCCTCGAGGGA GACAAGTAGAGATAGCTTTTTTTG; Reverse oligo: AATTCAAAAAAAGCTATCTCTACTTGTCTCCCTC GAGGGAGACAAGTAG AGATAGCTT) into pLKO.1 vector. Lentiviral particles were generated by transfecting 293T cells with packaging vectors, psPAX2 and pMD2.g. Medium was changed every 24 hours, the 48 and 72 hour supernatants were pooled, filtered through a 0.45 μm filter. Lentiviral particles were applied to C4-2 cells with 8 μg/mL polybrene (Sigma Aldrich), and infected cells were isolated by 10 μg/mL puromycin (Sigma Aldrich). SiRNA against CHIP and scramble siRNA (Santa cruz) were transfected into LNCaP cells using Lipofectamine 2000 (Invitrogen).

### Western blotting assay

Cells were treated with lysis buffer containing 150 mM Tris base (PH 7.5), 50 mM NaCl, 1 mM EDTA (PH 8.0), 1%NP40, 1 mg/ml leupeptin and 1 mM PMSF. Protein concentrations were determined by BCA protein assay (Pierce Chemical Co). Equal amounts of protein were separated by SDS-PAGE and blotted onto polyvinylidene fluoride membranes (Bio-Rad). After blocking with 5% non-fat milk in tris-buffered saline with tween at room temperature for 30 min, the membranes were probed with indicated antibodies. After a series of washes the blots were further incubated with goat anti-mouse or anti-rabbit IgG antibody conjugated to horseradish peroxidase (Zhong Shan Golden Bridge Biotechnology Co., LTD.) and detected using the ECL kit (Pierce).

### Co-immunoprecipitation assay

HEK293 cells were transiently transfected with the expression plasmids which are shown in the figures. After transfection 48 h, cells were lysed in lysis buffer (RIPA buffer) and 500 μl of crude cell lysate containing 200–500 μg of total protein were incubated with 20 μl anti-FLAG-M2 agarose beads. The immunoprecipitates were washed and resuspended in lysis buffer. The proteins bound to the agarose beads were analysed with the indicated antibodies for Western blotting. To test the endogenous AR interaction with CHIP, LNCaP and LNCaP-PC-1 cell lysates were incubated with 2 μg AR antibody and protein A/G-Sepharose beads (Santa Cruz Biotechnology) at 4°C overnight. Sepharose beads were washed with lysis buffer three times and resuspended in SDS-PAGE loading buffer for Western blotting using anti-CHIP antibody.

### Affinity purification of GST fusion proteins

*In vitro* expression and purification of recombinant GST fusion proteins were performed according to the protocol. Briefly, the recombinant vector was introduced into BL21 cells. An overnight culture of a colony of the transformants was diluted 1: 100 and grown for 1–2 h at 37°C before induction for 4–6 h at 30°C with 0.1 mM IPTG. The cultures were centrifuged at 4000 rpm for 5 min at 4°C and the cells were resuspended in ice cold phosphate-buffered saline (PBS). Bacterial cells were lysed by sonication on ice for 15 consecutive 15s intervals. Following addition of Triton X-100 to a final concentration of 1% (v/v) and centrifugation, the supernatants were incubated with glutathione-agarose beads (Amersham Biosciences) for 1 h at 4°C. Beads were used directly in GST-pull down assay after extensive washing with buffer.

### GST-pull down assay

For GST-pull down assay, HEK293 cells were transfected with the Myc-AR and AR deletion mutants or HA-PC-1 expression plasmid. The cells were rinsed with PBS and lysed in lysis buffer described above. Cell lysates were incubated with approximately equal amounts of GST or GST-PC-1/GST-CHIP fusion proteins at 4°C for 4–6 h with gentle rotation. The complexes were isolated by binding to glutathione-sepharose beads, and analyzed with the indicated antibodies for Western blotting.

### Ubiquitylation assay

HEK293 cells were co-transfected Myc-ubiquitin, Flag-AR/Flag-ARΔ629-634 with HA-PC-1 or Myc-CHIP alone or together. After transfection 36 h, cell lysates were immunoprecipitated with Flag beads and analyzed by Western blotting.

### Luciferase reporter assays

LNCaP, PC-3 and DU145 cells were seeded in 24-well plates in complete media and then transiently transfected with 300 ng of pARE_2_-Luc, or the PSA reporter plasmid, 500 ng-1000 ng of pcDNA3.1 or PC-1 expression plasmids, 25 ng pRL-TK (internal control) with or without 100 ng pSG5-hAR with the Lipofectamine2000 (Invitrogen). Complete medium was replaced after 36 h with cFBS with or without 1 nM R1881. The cells were lysed 48 h after transfection. Luciferase activity was subsequently measured using a Dual-Luciferase Assay kit (Promega) according to the manufacturer's instructions. All transfection experiments were carried out in triplicate wells and repeated three times.

### 3-(4,5-Dimethyl thiazol-2-yl)-2,5-diphenyl tetrazolium bromide (MTT) assay

Cell growth and viability was measured using the MTT proliferation assay. Briefly, 2000 cells were seeded in 96-well plates in 200 μl RPMI 1640 containing 10% FBS with or without 2 nM R1881 treatment. Cell growth was examined 7 days later. Before testing, 20 μl of MTT reagent (2.5 mg/ml MTT in PBS, Amresco Inc. Solon, Ohio) was added and the cells were incubated for a further 4 h at 37°C. Then 250 μl of dissolving reagent DMSO (Amresco Inc.) was added to dissolve the formazan crystals. The optical density (OD) was measured at wavelength of 490 nm on a microplate reader.

### Statistical methods

Statistics analysis was performed using two-tail Student's *t*-test. *p* < 0.05 was considered the threshold value for statistical significance.

## References

[1] Brinkmann AO, Klaasen P, Kuiper GG, van der Korput JA, Bolt J, de Boer W, Smit A, Faber PW, van Rooij HC, Geurts van Kessel A (1989). Structure and function of the androgen receptor. Urol Res.

[2] Chang CS, Kokontis J, Liao ST (1988). Structural analysis of complementary DNA and amino acid sequences of human and rat androgen receptors. Proc Natl Acad Sci USA.

[3] Shang Y, Myers M, Brown M (2002). Formation of the androgen receptor transcription complex. Mol Cell.

[4] Litvinov IV, Vander Griend DJ, Antony L, Dalrymple S, De Marzo AM, Drake CG, Isaacs JT (2006). Androgen receptor as a licensing factor for DNA replication in androgen-sensitive prostate cancer cells. Proc Natl Acad Sci. USA.

[5] Vander Griend DJ, Litvinov IV, Isaacs JT (2007). Stabilizing androgen receptor in mitosis inhibits prostate cancer proliferation. Cell Cycle.

[6] D'Antonio JM, Vander Griend DJ, Isaacs JT (2009). DNA licensing as a novel androgen receptor mediated therapeutic target for prostate cancer. Endocr Relat Cancer.

[7] Feldman BJ, Feldman D (2001). The development of androgen-independent prostate cancer. Nat Rev Cancer.

[8] Kokontis JM, Hsu S, Chuu CP, Dang M, Fukuchi J, Hiipakka RA, Liao S (2005). Role of androgen receptor in the progression of human prostate tumor cells to androgen independence and insensitivity. Prostate.

[9] Sharifi N (2013). Mechanisms of androgen receptor activation in castration-resistant prostate cancer. Endocrinology.

[10] Gioeli D, Ficarro SB, Kwiek JJ, Aaronson D, Hancock M, Catling AD, White FM, Christian RE, Settlage RE, Shabanowitz J, Hunt DF, Weber MJ (2002). Androgen receptor phosphorylation: Regulation and identification of the phosphorylation sites. J Biol Chem.

[11] Fu M, Wang C, Reutens AT, Wang J, Angeletti RH, Siconolfi-Baez L, Ogryzko V, Avantaggiati ML, Pestell RG (2000). p300 and p300/cAMP-response element-binding protein-associated factor acetylate the androgen receptor at sites governing hormone-dependent transactivation. J Biol Chem.

[12] Poukka H, Karvonen U, Janne OA, Palvimo JJ (2000). Covalent modification of the androgen receptor by small ubiquitin-like modifier 1 (SUMO-1). Proc Natl Acad Sci USA.

[13] Lin HK, Wang L, Hu YC, Altuwaijri S, Chang C (2002). Phosphorylation-dependent ubiquitylation and degradation of androgen receptor by Akt require Mdm2 E3 ligase. EMBO J.

[14] van der Steen T, Tindall DJ, Huang H (2013). Posttranslational modification of the androgen receptor in prostate cancer. Int J Mol Sci.

[15] He B, Bai S, Hnat AT, Kalman RI, Minges JT, Patterson C, Wilson EM (2004). An androgen receptor NH2-terminal conserved motif interacts with the COOH terminus of the Hsp70-interacting protein (CHIP). J Biol Chem.

[16] Rees I, Lee S, Kim H, Tsai FT (2006). The E3 ubiquitin ligase CHIP binds the androgen receptor in a phosphorylation-dependent manner. Biochim Biophys Acta.

[17] Li H, Xu LL, Masuda K, Raymundo E, McLeod DG, Dobi A, Srivastava S (2008). A feedback loop between the androgen receptor and a NEDD4-binding protein, PMEPA1, in prostate cancer cells. J Biol Chem.

[18] An J, Wang C, Deng Y, Yu L, Huang H (2014). Destruction of full-length androgen receptor by wild-type SPOP, but not prostate-cancer-associated mutants. Cell Rep.

[19] Qi J, Tripathi M, Mishra R, Sahgal N, Fazli L, Ettinger S, Placzek WJ, Claps G, Chung LW, Bowtell D, Gleave M, Bhowmick N, Ronai ZA (2013). The E3 ubiquitin ligase Siah2 contributes to castration-resistant prostate cancer by regulation of androgen receptor transcriptional activity. Cancer Cell.

[20] Wang R, Xu J, Saramaki O, Visakorpi T, Sutherland WM, Zhou J, Sen B, Lim SD, Mabjeesh N, Amin M, Dong JT, Petros JA, Nelson PS (2004). PrLZ, a novel prostate-specific and androgen-responsive gene of the TPD52 family, amplified in chromosome 8q21. 1 and overexpressed in human prostate cancer. Cancer research.

[21] Wang R, Xu J, Mabjeesh N, Zhu G, Zhou J, Amin M, He D, Marshall FF, Zhau HE, Chung LW (2007). PrLZ is expressed in normal prostate development and in human prostate cancer progression. Clinical cancer research.

[22] Zhang H, Wang J, Pang B, Liang RX, Li S, Huang PT, Wang R, Chung LW, Zhau HE, Huang C, Zhou JG (2007). PC-1/PrLZ contributes to malignant progression in prostate cancer. Cancer research.

[23] Haelens A, Tanner T, Denayer S, Callewaert L, Claessens F (2007). The hinge region regulates DNA binding, nuclear translocation, and transactivation of the androgen receptor. Cancer Res.

[24] Boutros R, Byrne JA (2005). D53 (TPD52L1) is a cell cycle-regulated protein maximally expressed at the G2-M transition in breast cancer cells. Exp Cell Res.

[25] WU Rui-Qin, LI Qi-Man, ZHANG Hui, YU Lan, ZHOU Jian-Cuang (2009). A new gene PC-1 of Tumor Protein D52 family expresses increasingly in G2/M Phase during cell cycle of prostate carcinoma cell lines LNCaP and C4-2B. Letters in Biotechnology.

[26] Zhang A, Zhao JC, Kim J, Fong KW, Yang YA, Chakravarti D, Mo YY, Yu J (2015). LncRNA HOTAIR Enhances the Androgen-Receptor-Mediated Transcriptional Program and Drives Castration-Resistant Prostate Cancer. Cell Rep.

[27] Clinckemalie L, Vanderschueren D, Boonen S, Claessens F (2012). The hinge region in androgen receptor control. Mol Cell Endocrinol.

[28] Hörnberg E, Ylitalo EB, Crnalic S, Antti H, Stattin P, Widmark A, Bergh A, Wikström P (2011). Expression of androgen receptor splice variants in prostate cancer bone metastases is associated with castration-resistance and short survival. PLoS One.

[29] Hu R, Lu C, Mostaghel EA, Yegnasubramanian S, Gurel M, Tannahill C, Edwards J, Isaacs WB, Nelson PS, Bluemn E, Plymate SR, Luo J (2012). Distinct transcriptional programs mediated by the ligand-dependent full-length androgen receptor and its splice variants in castration-resistant prostate cancer. Cancer Res.

[30] Li Y, Chan SC, Brand LJ, Hwang TH, Silverstein KA, Dehm SM (2013). Androgen receptor splice variants mediate enzalutamide resistance in castration-resistant prostate cancer cell lines. Cancer Res.

[31] Dehm SM, Tindall DJ (2011). Alternatively spliced androgen receptor variants. Endocr Relat Cancer.

[32] Sarkar S, Brautigan DL, Parsons SJ, Larner JM (2014). Androgen receptor degradation by the E3 ligase CHIP modulates mitotic arrest in prostate cancer cells. Oncogene.

[33] Linn DE, Yang X, Xie Y, Alfano A, Deshmukh D, Wang X, Shimelis H, Chen H, Li W, Xu K, Chen M, Qiu Y (2012). Differential regulation of androgen receptor by PIM-1 kinases via phosphorylation-dependent recruitment of distinct ubiquitin E3 ligases. J Biol Chem.

[34] Haile S, Sadar MD (2011). Androgen receptor and its splice variants in prostate cancer. Cell Mol Life Sci.

[35] Niu Y, Chang TM, Yeh S, Ma WL, Wang YZ, Chang C (2010). Differential androgen receptor signals in different cells explain why androgen-deprivation therapy of prostate cancer fails. Oncogene.

[36] Niu Y, Altuwaijri S, Lai KP, Wu CT, Ricke WA, Messing EM, Yao J, Yeh S, Chang C (2008). Androgen receptor is a tumor suppressor and proliferator in prostate cancer. Proc Natl Acad Sci USA.

[37] Carver BS, Chapinski C, Wongvipat J, Hieronymus H, Chen Y, Chandarlapaty S, Arora VK, Le C, Koutcher J, Scher H, Scardino PT, Rosen N, Sawyers CL (2011). Reciprocal feedback regulation of PI3K and androgen receptor signaling in PTEN-deficient prostate cancer. Cancer Cell.

[38] Li L, Xie H, Liang L, Gao Y, Zhang D, Fang L, Lee SO, Luo J, Chen X, Wang X, Chang LS, Yeh S, Wang Y (2013). Increased PrLZ-mediated androgen receptor transactivation promotes prostate cancer growth at castration-resistant stage. Carcinogenesis.

[39] Yu SQ, Han BM, Shao Y, Wu JT, Zhao FJ, Liu HT, Sun XW, Tang YQ, Xia SJ (2009). Androgen receptor functioned as a suppressor in the prostate cancer cell line PC3 *in vitro* and *in vivo*. Chin Med J (Engl).

[40] Litvinov IV, Antony L, Dalrymple SL, Becker R, Cheng L, Isaacs JT (2006). PC3, but not DU145, human prostate cancer cells retain the coregulators required for tumor suppressor ability of androgen receptor. Prostate.

